# Tuberculous lumbar spinal epidural abscess in a young adult (case report)

**DOI:** 10.1051/sicotj/2018005

**Published:** 2018-03-09

**Authors:** Ghazwan Abdulla Hasan, Saif Mohammed Kani, Ahmed Alqatub

**Affiliations:** Medical City Complex, Baghdad Iraq

**Keywords:** Tuberculosis, Spinal epidural abscess, Lumbar spine

## Abstract

*Introduction*: Spinal Epidural abscess (SEA) is an uncommon pathology that needs an urgent intervention to decompress the pressure on the spinal epidural sac, cord, and roots. The authors report a rare case of a young adult with lumbar spinal epidural tuberculous abscess occupying the spinal canal from L2–L5 vertebrae with extesion to the posterior paraspinal muscles and presenting with bilateral progressive lower limb weakness.

*Case report*: A 42 years old male teacher presented with a 15-day history of progressive difficulty to walking and bilateral lower limb weakness associated with fever, malaise and later on urinary incontinence. A magnetic resonance imaging (MRI) scan revealed a paraspinal intermuscular abscess and an abscess occupying the spinal canal compressing the dural sac from L2–L4/5, without any signs of vertebral involvement. Surgery was done by a posterior midline incision. Pus was evacuated from multiple pockets through the paraspinal muscle layers. Laminectomy for L3/4, and hemilaminectomy for L2/3, and L4/5 were performed. Pus and bone specimens were negative for acid-fast bacilli. However, both histopathological studies and Polymerase Chain Reaction (PCR) testing confirmed the presence of tuberculosis (TB). The patient received TB antibiotics, and a follow-up MRI scan at 2 months showed complete evacuation of the abscess. However, signs of L5 spondylitis were evident. No further surgery was needed as there was no vertebral collapse or neural compression and the patient's clinical condition was improving. He had normal right lower limb power and sensation and grade 4+ motor power of the left lower limb. Bowels and bladder function was normal.

*Conclusion*: Isolated tuberculous spinal epidural abscess is a rare disease and should be treated urgently with evacuation and decompression. Signs of spondylitis or spondylodiscitis may appear later and therefore long follow up is recommended in tuberculous cases presenting with an isolated epidural abscess.

## Case presentation

A 42 years old male teacher referred to our hospital complaining of the 15-day history of progressive back pain radiating to both thighs. He subsequently developed weakness affecting both lower limbs, but mainly on the left side, with urinary incontinence and constipation. It was associated with low-grade fever, loss of appetite and ended with the inability to walk.

On examination, he looked pale and uncomfortable. He had a pulse rate of 95 beats per minute, blood pressure 110/70, temperature 38.3 °C, and a respiratory rate of 19 cycles per minute.

### Musculoskeletal examination

Inability to walk, severe tenderness in the back around the midline and paraspinal region, and severe limitation of spinal motion.    

### Neurological examination

*Sensation:*  paresthesia below the inguinal ligaments in both lower limbs.

### Motor

Left side:

- grade 2 knee extension;
- grade 1 knee flexion;
- grade 1 ankle dorsiflexion;
- grade 1 ankle plantar flexion.

Right side:

- grade 3 knee extension and flexion;
- grade 2 ankle plantar flexion and dorsiflexion.

#### Reflexes:

- diminished knee and ankle jerk;
- equivocal Babiniski sign.

#### Sphincters:

- urinary incontinence;
- constipation.

### Laboratory analyses

Hemoglobin 11.1 g/dL, white blood cell count 11 × 10^9^ with lymphocytosis (60%), essential sedimentation rate 95 mm/hour, C-reactive protein 90, random blood sugar 114 g/dL, blood urea 78 mg/dL, and serum creatinine 1.3.

### Imaging study

**Figure 1:**
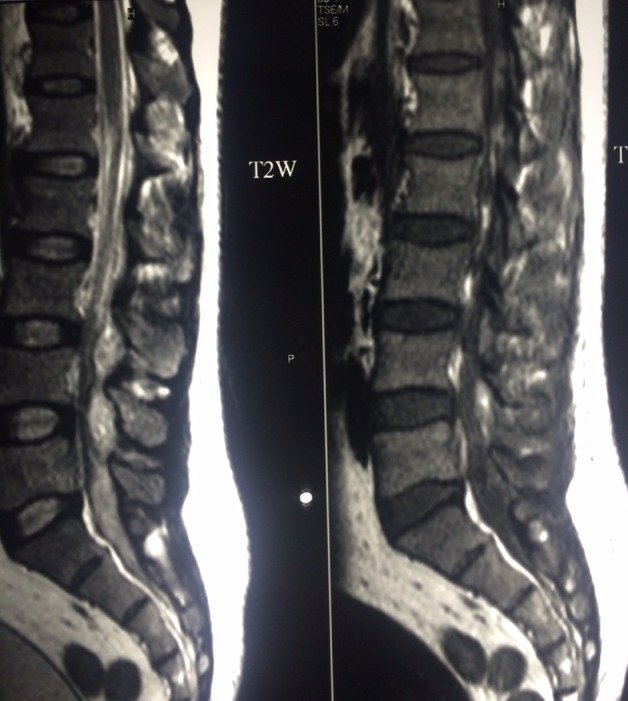
Preoperative MRI sagittal section, T2W (left picture), T1W (right picture) show localized collection of pus in the canal at the level of L3–4 with compression on the dural sac.

**Figure 2:**
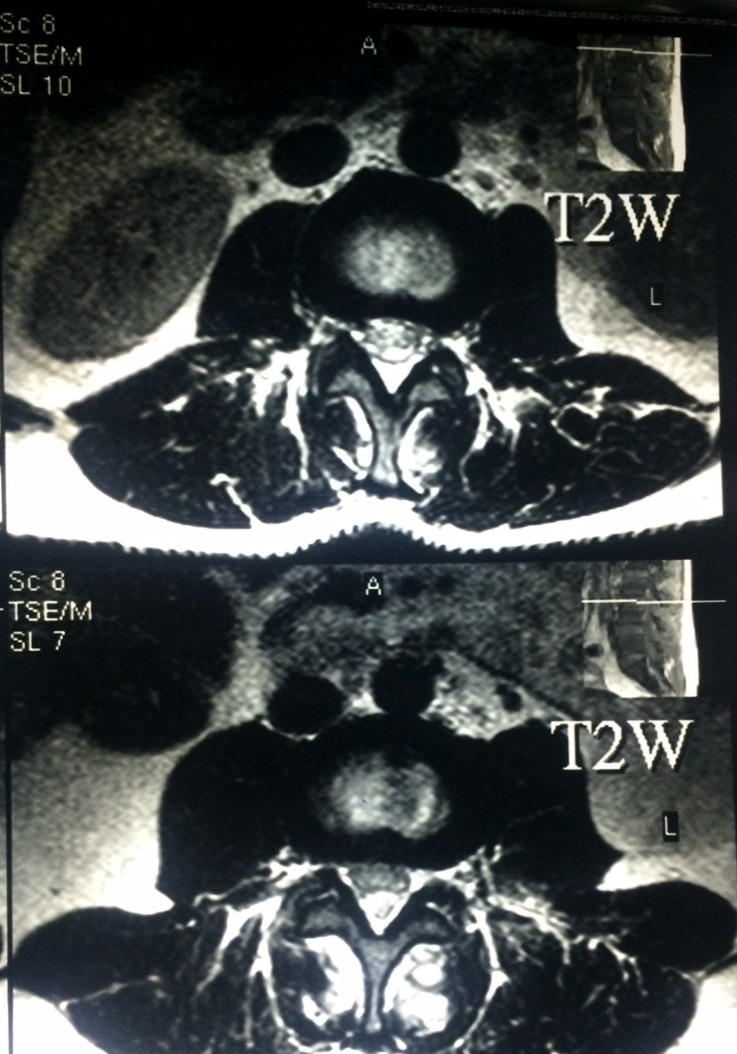
Preoperative MRI, axial section T2W  at the level of L3 & L4 show canal compression on dural by pus collection.

**Figure 3:**
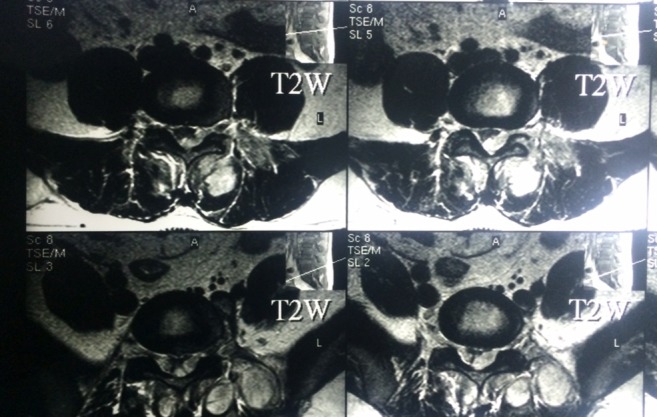
Preoperative MRI, axial section T2W, show multiple pockets of pus in the paraspinal muscles.

Plain X-ray of the lumbosacral spine in both Antero-posterior and lateral view revealed no abnormality. MRI of lumbosacral spine in both T1, T2, and STIR showed a localized collection of pus in the canal compressing the dural sac at L3, L4 and L5 levels with a small collection of pus at L2–L3 level. Also, there were multiple pockets of pus in the paraspinal muscles (Figures 1–3).  

## Procedure

After discussing the treatment options with the patients, we decided to proceed with surgery. Using a posterior midline approach, in a prone position and under general anesthesia, the paraspinal muscles were dissected. We noticed multiple pockets of pus imbedded in the paraspinal muscles, mainly on the left side, which were evacuated. Then a laminectomy of L3–L4 was done with hemilaminectomy at L2–L3 and L4–L5. Pus was draining from these levels through the spinal canal. Samples were taken from pus, laminae bone, and soft tissue and sent for histopathological and microbiological studies.

Thorough irrigation and debridement were done, a drain was applied, and the wound was closed in layers.

The patient was kept on broad-spectrum antibiotics for 14 days pending microbiological laboratory results.

### Sample results

Microbiological results showed no growth of bacteria, Acid Fast Bacilli was negative, but Polymerase chain reaction (PCR) was positive.

Histopathological study: sections showing calcified mature bony trabeculae and degenerative cartilaginous tissues component in between marked hematopoietic depletion, dense chronically inflamed fibrous replacement including heavy mixed chronic inflammatory cells which include foamy histiocytic and mature lymphocytic cells infiltrate. Multiple ill-defined granulomas were seen with obvious extra bony inflammatory extension to the surrounding soft tissue and skeletal, muscular tissues. The final impression was a chronic specific tuberculous granulomatous spinal cold abscess.

## Follow up and outcome

Anti TB antibiotics were started after the above results became available.

Postoperatively, gradual neurological recovery occurred in both lower limbs. At one month follow up, the right lower limb had full motor recovery (grade 5), while the left lower limb was weaker on knee flexion and ankle dorsiflexion (grade 4). The patient was still incontinent to urine and constipated.

**Figure 4:**
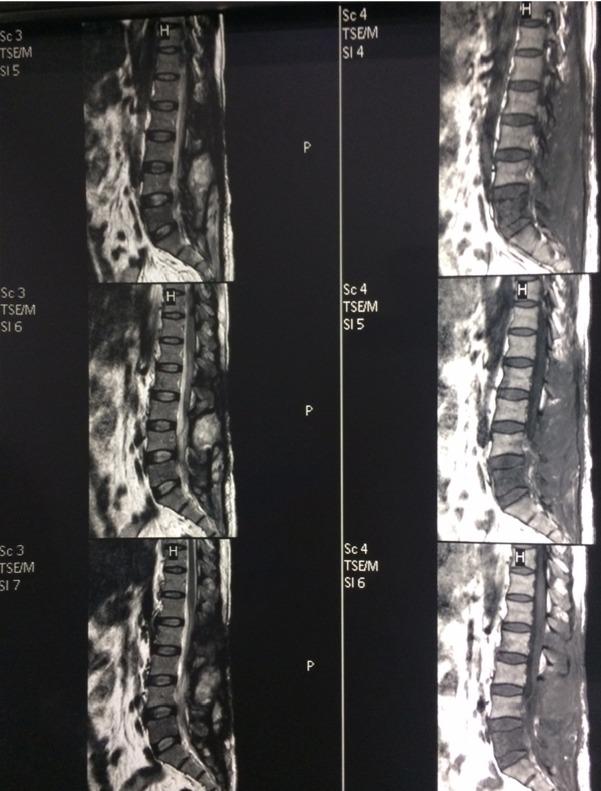
Two-month follow-up MRI showing complete abscess resolution and neural decompression but low signal of L5 in T1 (a) and high signal inT2 (b) indicating L5 spondylitis.

At two months after surgery, the left lower limb showed further neurological improvement (grade 4+), and though the patient reported some improvement of his sphincter control, he was still complaining of nocturnal incontinence and constipation. A follow-up MRI at two months of index surgery showed complete abscess resolution with good decompression of the cauda equina region (Figure 4). Surprisingly, The images also showed signs of spondylitis of L5. Because the clinical condition of the patient was improving, there were no signs of discitis nor vertebral collapse, the patient was further treated conservatively.

Five months later, he recovered both lower limbs motor strength and full control of his sphincters.

## Discussion

Vertebral TB accounts for less than 1% of all TB infections in the body and more than 50% of musculoskeletal infections. It is considered the most serious type of skeletal TB, with possible neurological symptoms due to compression of the neural structures. It may also lead to deformity and significant vertebral structure destruction and instability [1]. A spinal epidural abscess is a rare disease with a reported incidence of 1–2/10 000; TB accounts for 2% of these infections [2].

Cases of the SEA due to TB infection have been reported in developing countries. The clinical presentation of the tubercular SEA may be more insidious, leading to failure of early diagnosis treatment [3].

Initial presentation of the tubercular SEA may include back pain, low-grade fever, and severe neurological deficit. The classical triad of fever, back pain and neurological deficits present in only 2% of the tubercular SEA [2]. Thoracic and lumbar region are the most commonly affected regions [4]. MRI is the diagnostic modality of choice. CT myelogram can be done for those who cannot undergo MRI [5].

There are only a few reported cases of the tubercular SEA. Most of these infections are secondary to pyogenic infection [6–8].

Treatment options depend on many factors like the site and severity of compression, and clinical presentation. Medical treatment can be offered in certain cases of the tubercular SEA. Surgery is indicated when there is compression of neurological structures, failure of resolution, doubtful diagnosis, and other reasons like mechanical instability. The options for surgical treatment may vary from decompression laminectomy, multilevel laminotomy or laminectomy with or without fixation; all this can be determined by the adequacy of decompression intraoperatively [1].

Infection of the spine may lead to wide range of problems including the neurological deficit, mechanical instability, and deformity [3]. Very few isolated tubercular spinal epidural abscess have been reported [3,4,9] because most of the epidural abscesses are secondary to pyogenic infection [6–8]. Tubercular SEA usually develops secondary to involvement of vertebral body and rarely by hematogenous spread from a primary focus in the body [9–11].

Involvement of vertebral body of the lumbar spine in T1W follow up MRI with low signal intensity could be due to direct spread from the abscess or a reactivation of a dormant focus. The clinical presentation in our patient suggested compression in the cauda equine region with the highest level of involvement at L2.

Tubercular SEA is amenable to both non-surgical and surgical management [3]. In our case, we chose the surgical treatment because of the progressive neurological deficit. Kennedy et al. [12] concluded that early diagnosis and decompression are the most important predictive parameters of favorable outcomes [3,12]. We decompressed the cauda equina by performing laminectomy at the level of L3–L4, and hemilaminectomy at L2–L3; and L4–L5 levels to relieve the clinically evident compression. The evacuation of pus irrigation of the area was done including multiple pockets of pus collection through the paravertebral muscles.

## Conclusion

This case report highlights the importance of early diagnosis and early treatment of tuberculous SEA. Isolated tuberculous epidural abscess is rare and can precede the development of tuberculous spondylitis, which mandates long follow up.

## Conflict of interest

The authors declare that they have no conflicts of interest in relation to this article.
